# Collaborative Health and Enforcement Operations on the Quality of Antimalarials and Antibiotics in Southeast Asia

**DOI:** 10.4269/ajtmh.14-0574

**Published:** 2015-06-03

**Authors:** Yuk Lin Yong, Aline Plançon, Yen Hui Lau, Dana M. Hostetler, Facundo M. Fernández, Michael D. Green, Sourisak Sounvoravong, Suon Nara, Mam Boravann, Thitikornkovit Dumrong, Nurjaya Bangsawan, Min Yong Low, Chin-Chin Lim, Ruth Lee Choo Ai, Paul N. Newton

**Affiliations:** Health Sciences Authority, Singapore; Medical Product Counterfeiting and Pharmaceutical Crime Sub-Directorate, INTERPOL, Lyon, France; School of Chemistry and Biochemistry, Georgia Institute of Technology, Atlanta, Georgia; Centers for Disease Control and Prevention (CDC), Atlanta, Georgia; Food and Drug Department, Ministry of Health, Government of the Lao PDR, Vientiane, Lao PDR; Anti-Economic Crime Department, Cambodian National Police, Royal Government of Cambodia, Cambodia; Department of Drugs and Food, Ministry of Health, Royal Government of Cambodia, Cambodia; Food and Drug Administration, Bangkok, Kingdom of Thailand; Food and Drug Administration, Jakarta, Republic of Indonesia; Lao-Oxford-Mahosot Hospital-Wellcome Trust Research Unit, Microbiology Laboratory, Mahosot Hospital, Vientiane, Lao PDR; Centre for Tropical Medicine and Global Health, Nuffield Department of Medicine, Churchill Hospital, Oxford University, England, United Kingdom; Worldwide Antimalarial Resistance Network, Churchill Hospital, Oxford University, England, United Kingdom; London School of Hygiene and Tropical Medicine, London, England, United Kingdom

## Abstract

Counterfeit (or falsified) and substandard medicines pose a major public health risk. We describe the findings of Operation Storm I and II conducted in 2008–2009 to combat counterfeit medicines through partnership between national customs, Drug Regulatory Agencies (DRAs), and police in Cambodia, Indonesia, Laos, Myanmar, Singapore, Thailand, and Vietnam. Samples were obtained from seizures and market surveillance by national DRAs. Laboratory analysis using spectroscopic and chromatographic techniques and examination of packaging were performed. Ninety-three suspect antibiotics and 95 antimalarial samples were collected. Of the 93 antibiotics, 29 (31%) had % active pharmaceutical ingredient content (%API) < 85% or > 115% (including one counterfeit). Of the 95 antimalarials, 30 (32%) had %API < 85 > 115% API (including one counterfeit). A significant minority of samples, antimalarials (13%) and antibiotics (15%), were collected in plastic bags with minimal or no labeling. Of 20 ampicillin samples, 13 (65%) contained < 85% API (with one counterfeit containing additional amoxicillin). Of 34 oral artesunate samples, 7 (21%) contained %API out of the 85–115% range. Coordinated and synergistic partnership adopted by the participating countries, International Criminal Police Organization (INTERPOL), World Health Organization (WHO), and laboratories facilitated a platform for discussions and intelligence sharing, helping to improve each participating country's capacity to combat poor-quality medicines.

## Introduction

There is an increasing concern that the supply of essential medicines (WHO 2010) in financially poor countries is contaminated with counterfeit and substandard medicines.^1^–^16^ Such “medicines” increase mortality and morbidity, engender drug resistance, and cause economic losses for patients, health systems, and the pharmaceutical industry.

Building on Operation Jupiter,^8^ an operation to investigate falsified artesunate in southeast Asia, International Criminal Police Organization (INTERPOL), together with the World Health Organization (WHO) set up Operation Storm in the Greater Mekong region of southeast Asia. Operation Storm I and II were conducted by seven participating countries (Cambodia, Indonesia, Lao PDR, Myanmar, Singapore, Thailand, and Vietnam) between April 2008 and November 2009. This multi-country operation provided a common platform for coordination meetings and training. Three national agencies, the customs, Drug Regulatory Agencies (DRAs), and the police in each participating country conducted joint operations to investigate suspected pharmaceutical crime.

There has been confusion and controversy over the definitions of counterfeit and substandard medicines. We use the term counterfeit medicines to describe those “…deliberately and fraudulently mislabeled with respect to identity and/or source.” Counterfeiting can apply to both innovative and generic products and may include products with the correct ingredients, wrong ingredients, without active ingredients, with insufficient quantity of active ingredient, or with fake packaging.^17^ The term counterfeit medicine was generally accepted when first used in 1988 by WHO, but is now increasingly perceived as associated with intellectual property rights, rather than with public health. It has been proposed to replace “counterfeit” in WHO's definition with “falsified,” and to reserve the term “counterfeit medicine” for a “falsified” medicine with a “counterfeit” trademark.^18^ In this paper, as the participating countries have not adopted the term “falsified,” we continue to use the term “counterfeit” by WHO's definition, but devoid of intellectual property implications. Substandard medicines are “genuine medicines produced by manufacturers authorized….which do not meet quality specifications set for them by national standards.”^3^,^18^–^20^ Subtherapeutic amounts of active pharmaceutical ingredients (APIs) and/or suboptimal release of API (dissolution) are often found in substandard medicines and sometimes in counterfeit medicines, exposing parasites to sublethal concentrations of the APIs, hence engendering drug resistance.^11^

There is evidence that counterfeit/substandard essential medicines are important public health problems in southeast Asia.^2^,^9^,^21^ To investigate the quality of medicines and foster collaboration between DRAs, police, and customs, suspect medicines were collected by seizures and market surveillance. We present the laboratory findings and discuss their regulatory and public health implications.

## Materials and Methods

### Samples.

Samples were collected by the national DRA inspection staff in the seven participating countries (Cambodia, Indonesia, Lao PDR (Laos), Myanmar (Burma), Singapore, Thailand, and Vietnam) and submitted to the Health Sciences Authority (HSA), Singapore, Center for Disease Control and Prevention (CDC), Atlanta, GA, and the Georgia Institute of Technology (GT), Atlanta, GA for chemical analysis. Samples were collected for Storm 1 between April 2008 and March 2009 and Storm II between July and November 2009.

We define a sample as a group of apparently physically identical dosage units (e.g., tablets or vials) with the same batch number and brand name obtained at the same time from the same outlet. DRAs were requested by INTERPOL to focus on four classes of essential medicines (antimalarials, antibiotics, anti-HIV, and anti-tuberculosis). The findings discussed here are related to the antibiotics and antimalarials collected. The aim of this work was not to estimate the prevalence of counterfeit and substandard medicines in the sampled countries,^22^ but to pilot collaborative investigations between DRAs, police, and customs in identifying medicine quality problems and build these linkages. Samples were given unique identifiers and information on sampling location and type of outlet, submitter's details, manufacturing information (manufacturer, manufacturing and expiry dates, batch numbers), and the chemical and packaging analysis results were recorded.

### Authentic reference samples.

Thirty-nine authentic reference samples were provided by the Cambodia Drug Regulatory Organisation, Vietnam Drug Quality Management Division, Sanofi Synthelabo Viet Nam, Eli Lilly and Company, Mekophar Chemical Pharmaceutical Joint-Stock Company, Guilin Pharmaceutical Co. Ltd, and Kunming Pharmaceutical Corp. Of these samples, 2 were antibiotics and 18 were antimalarials.

### Chemical and packaging analysis.

Chemical and packaging analysis of all samples was performed at HSA except for the antimalarials collected in Operation Storm II, which were analyzed at CDC and GT.

### Chemical analysis.

The samples received by HSA were analyzed for their chemical composition, including the active pharmaceutical ingredients (API). Coatings and cores of the tablets, capsule shells, and contents were analyzed by Fourier transform infrared spectroscopy (FTIR) (Nicolet 380 with Centaurus Microscope, Thermo Scientific, Waltham, MA) and Raman spectroscopy (RM1000, Renishaw, Wotton-under-Edge, Gloucester, UK; DXR Smart Raman and DXR Raman Microscope, Thermo Scientific). High-performance liquid chromatography (HPLC) with UV detection (Agilent 1200 HPLC, Agilent, Santa Clara, CA) was used to determine %API using methods described in the United States Pharmacopoeia (USP), British Pharmacopoeia (BP), or International Pharmacopoeia (IP). The number of tablets or capsules used for chemical assay followed the European Network of Forensic Science Institutes (ENFSI) drug sampling guidelines (United Nations Office on Drugs and Crime [UNODC] with ENFSI Drugs Working Group, 2009).^23^ The tablets were ground and the capsule contents were mixed and homogenized. A composite of the ground tablets or mixed capsule contents was analyzed in duplicate.

At CDC, antimalarials and co-trimoxazole antibiotics were analyzed by a modified HPLC protocol.^24^ Briefly, all tablets were pulverized, dissolved in the appropriate solvent, and filtered using a 0.22 μm nylon membrane. A portion of the extract was injected into the HPLC system. Component separation was achieved using a 150 × 4.6 mm octadecylsilica column and a mobile phase consisting of various proportions of acetonitrile and 0.05 M perchlorate buffer adjusted to a pH of 2.5 with UV detection. In addition, two different direct ionization mass spectrometry (MS) methods were used: Direct Analysis in Real Time (DART) (DART-100, IonSense, Saugus, MA) and Desorption Electrospray Ionization-MS (DESI) (custom built) in both conventional and reactive modes.^25^,^26^ Four dosage units (individual tablets or capsules) were examined for each sample, unless fewer than 4 units were submitted. In both laboratories, the primary reference range for %API used was 85–115% but we also express the results in the more stringent %API 90–110% limits.

### Packaging analysis.

Packaging analysis was conducted at HSA and at the Lao-Oxford-Mahosot Hospital Wellcome Trust Research Unit (LOMWRU). At HSA, the printing quality and defects, and security features of the packaging (carton, blister pack, leaflet insert), tablets and capsules, and the debossing marks of batch numbers, manufacturing and expiry dates on cartons, blister packs and tablets were examined and compared with authentic reference samples, if available, using a comparison microscope and a forensic light source with light of varying wavelengths (405–525 nm). Batch numbers, manufacturing and expiry dates, color, dimensions, prints, defects, and marks of the packaging, tablets, and capsules were documented. Similar analysis was performed at LOMWRU except that a light source of 375 nm was used and debossing marks were not analyzed.

### Interpretation and classification.

Authentic reference samples are important for classification of a sample as “counterfeit,” “substandard,” or “authentic.” Using WHO's definition, we classified a medicine as “counterfeit”(approximating falsified) if the dosage form and/or packaging was falsified. The dosage form was considered counterfeit if it contained the wrong type of API or no API. The packaging was considered counterfeit if the type and quality of printing, or security features differ from that of the authentic reference packaging. Substandard medicines were classified as those that had authentic packaging but with stated API outside pharmacopeial limits. Of note, substandard medicines could not be distinguished from degraded medicines.^27^

We requested DRAs and manufacturers for reference samples of authentic products. If available, the authenticity of a suspect sample could be determined, and counterfeit and substandard samples distinguished. If the authentic reference sample was unavailable, we classified suspect samples according to their %API, primarily as poor quality if outside the range 85–115% and also secondarily if outside the range 90–110% API. The results have been reported using the MEDQUARG guidelines where possible.^22^

## Results

Ninety-three antibiotic samples and 95 antimalarial samples, comprising a median (range) of 1 (1–5) and 1 (1–12) dosage units, respectively, were collected (Table 1 and Supplemental Table 1).

### Antibiotics.

The 93 antibiotic samples, collected in Cambodia, Laos, and Thailand, contained 11 stated APIs: amoxicillin, ampicillin, chloramphenicol, ciprofloxacin, cloxacillin, doxycycline hyclate, erythromycin, metronidazole, phenoxymethylpenicillin (penicillin V, Pen-V), sulfamethoxazole-trimethoprim, and tetracycline. Only two reference amoxicillin samples, “AMOX-MS” manufactured by Medical Supply Pharmaceutical Enterprise and “Amoxicillin” manufactured by Bailly-Creat, were available for comparison.

Fifty-four (58%) antibiotics were classed as having %API within the 90–110% range (including three with authentic packaging available for comparison) and one had %API > 115%, 28 had %API < 85% (including one counterfeit), and an additional 10 had %API between 85% and < 90%. Therefore, using the %API < 85 > 115% limits, 29 (31%) failed and using the more stringent criteria of %API < 90 > 110%, 39 (42%) failed.

As we were only able to obtain two reference samples for comparison, we cannot be certain whether most failed samples were counterfeit or substandard. Fourteen (15%) of the antibiotics collected were in plastic bags without labeling as to API content, batch number and/or manufacturer information (Cambodia–Pen V–1, Cambodia–tetracycline–1, Laos–ampicillin–1, Laos–tetracycline–1, Thailand–Pen V–3, Thailand– tetracycline–7).

**Figure 1.**
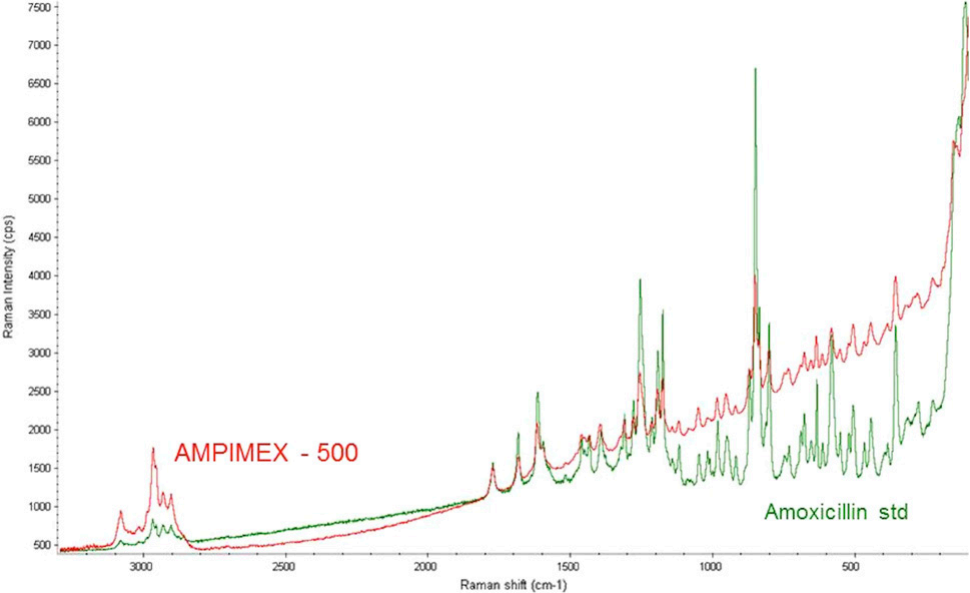
Raman spectra of an ampicillin sample labeled as “AMPIMEX-500” (red) and an amoxicillin standard (green). Amoxicillin, not stated in the label of “AMPIMEX-500,” was detected in the Raman spectrum of the capsule content.

Of the 20 ampicillin samples, 13 (65%) samples contained < 85% of the stated ampicillin content, with 3 samples containing API < 50%. One ampicillin sample, labeled as “AMPIMEX-500,” stated to contain 500 mg ampicillin, and labeled as manufactured by “Fu Li Pharmaceutical China,” was classed as counterfeit. Not only did the sample contain only 15% of the stated API (76.7 mg/cap of ampicillin), it also contained amoxicillin (66.6 mg/cap), an API which was not specified on the packaging. The presence of amoxicillin in the sample was easily identified by Raman spectroscopy (Figure 1).

**Figure 2.**
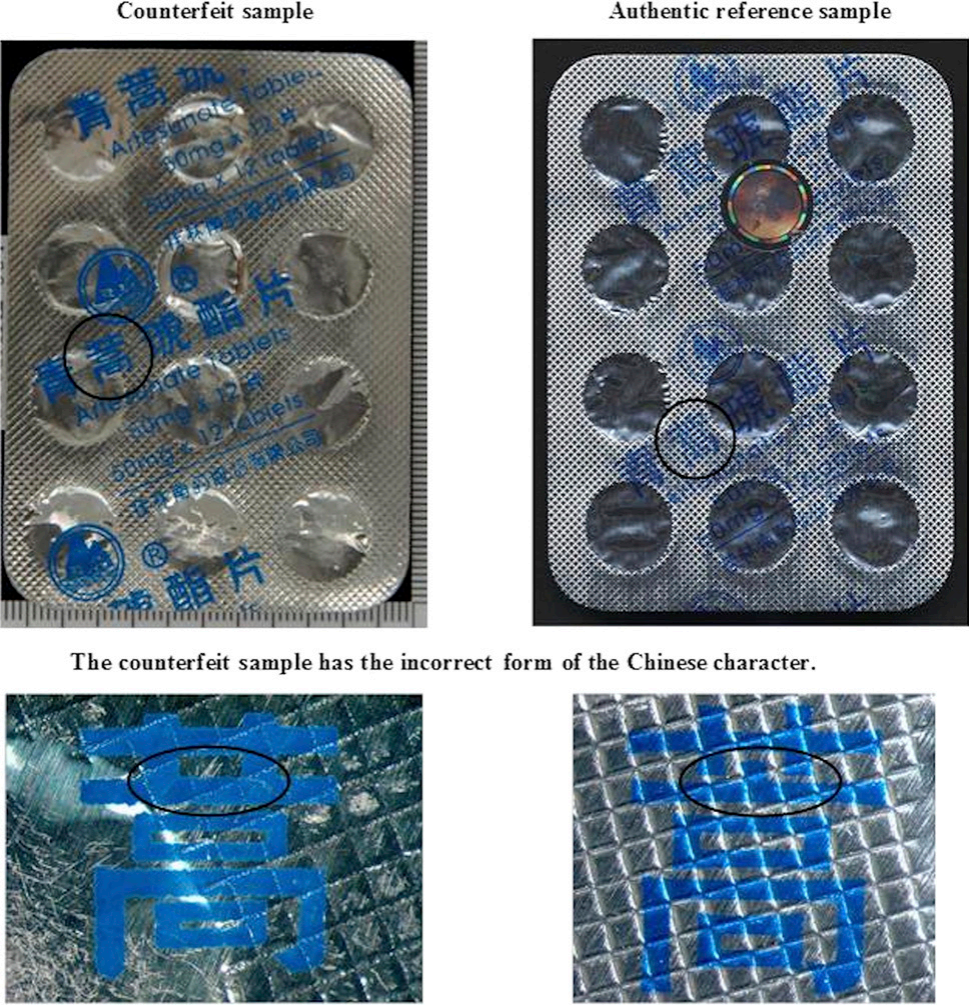
Magnified views of the numerals and characters of the lot number and expiry date: “87,” “X,” and “11,” on the blister packs of the suspect sample “AMOX-MS (500 mg)” and the authentic reference sample.

Four amoxicillin samples, including two labeled as manufactured by “Baily-Creat” and two labeled as “AMOX-MS” manufactured by “Medical Supply Pharmaceutical Enterprise,” were classified as authentic. The “AMOX-MS (500 mg)” sample bore similar machine marks on the embossed expiry date and lot number on the blister pack as those on the blister pack of the authentic reference sample, suggesting that they were manufactured on the same machine (Figure 2).

Three manufacturers—“Laboratories EPHAC Co.,” “Glenmark,” and “Shijiazhuang Ouyi Pharmaceutical” were stated on the labeling as the producers of products that on analysis contained unacceptable %API. Amoxicillin, ampicillin, and tetracycline products labeled as from the former two manufacturers contained lower than the stated API, at 70 to < 90% of the amounts stated on the packaging. Amoxicillin and ampicillin were present at only 40–50% of the stated amounts for the samples labeled as from “Shijiazhuang Ouyi Pharmaceutical.” As we were not able to obtain authentic reference samples from these manufacturers, we could not ascertain whether these products were counterfeit.

### Antimalarials.

The 95 antimalarial samples, collected in Cambodia, Indonesia, Laos, Myanmar, Thailand, and Vietnam, contained 9 different APIs, including monotherapies (artesunate, artemether, chloroquine, mefloquine, and quinine) and co-formulated/co-blistered combination therapies (dihydroartemisinin [DHA]-piperaquine phosphate, sulfadoxine-pyrimethamine, artesunate + amodiaquine, and artesunate + mefloquine).

Fifty-eight (61%) of the 95 antimalarial samples were classed as having %API within the 90–110% range (with 18 having authentic packaging for comparison); 1 was counterfeit, 5 (5%) had %API > 115%, with an additional 2 with API > 110%, 25 (26%) had %API < 85% with an additional 5 (5%) having API < 90%. Therefore, using the %API < 85 > 115% limits, 30 (32%) failed and using the more stringent criteria of %API < 90 > 110%, 37 (39%) failed.

Twelve (13%) of the antimalarial samples collected were in plastic bags without labeling as to API, batch number, and/or manufacturer information (Cambodia chloroquine-18, Vietnam chloroquine-1, Vietnam quinine-1, Lao quinine-10, Thailand chloroquine-2, Thailand quinine-1). The 34 oral artesunate monotherapy samples were labeled as manufactured by 8 different companies with 10 different types of packaging. Seven (21%) contained %API outside the 85–115% range with an additional 3 (15%) outside the 90–110% range. Co-formulated DHA-piperaquine from Cambodia bore the text “2 days malaria treatment” on the packaging.

**Figure 3.**
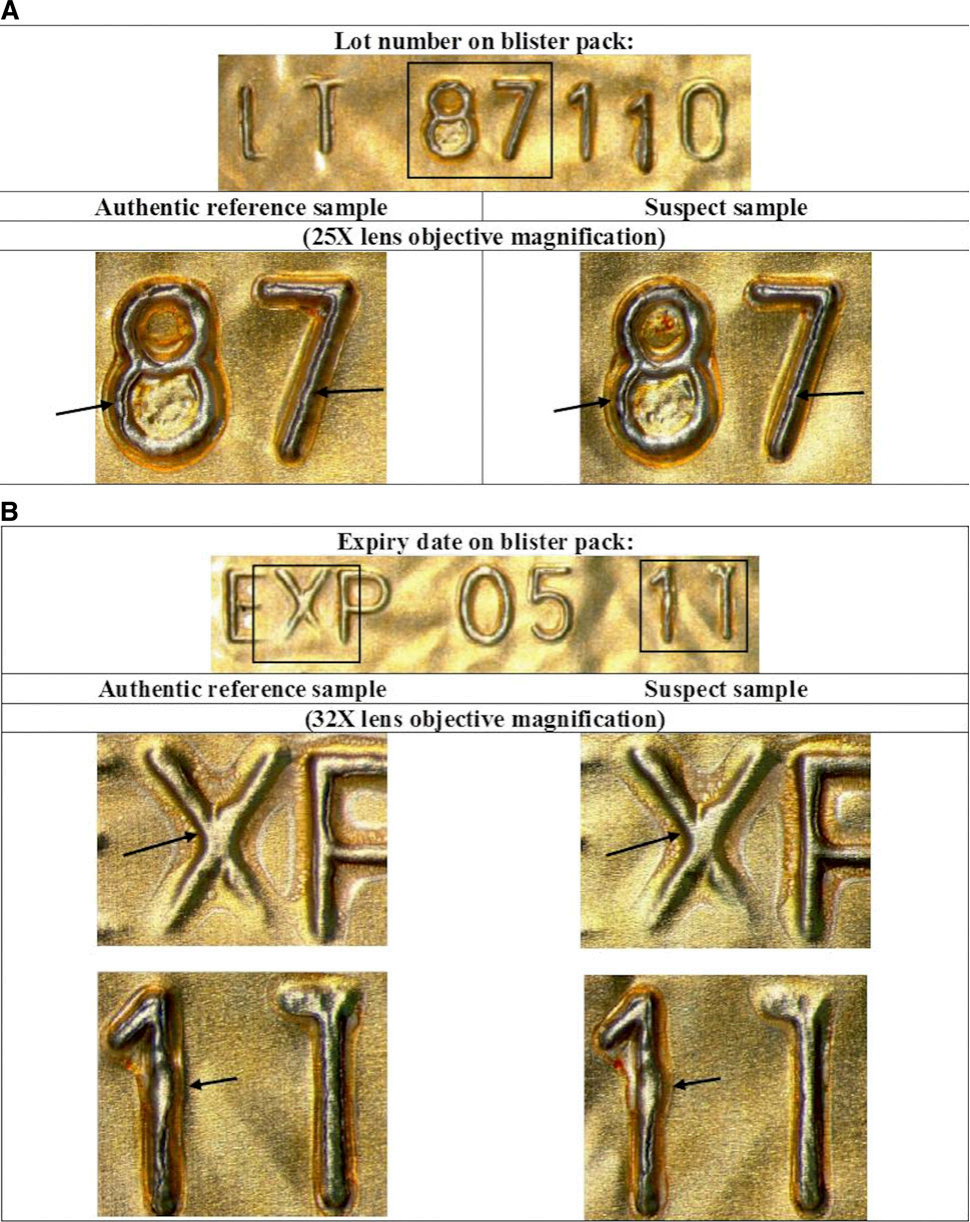
(**A** and **B**) Scans of counterfeit artesunate sample labeled as manufactured by “Guilin Pharmaceutical Co. Ltd,” in comparison to an authentic standard demonstrating error in Chinese character.

Two artesunate samples were collected in Laos, labeled as manufactured by “Mekophar” with one containing 86% and the other 144% of the dose of artesunate stated on the blisterpack. Although the print designs on the blisterpacks were different from the authentic reference samples, we were unable to determine if the design was a legitimate variation from Mekophar. One “artesunate” sample collected in Laos, labeled as manufactured by “Guilin Pharmaceutical Co. Ltd,” contained no detectable artesunate, but mainly calcium carbonate, as determined by FTIR, Raman spectroscopy, and HPLC assays. Inspection of the blister packaging (Figure 3A and B) indicated that these were counterfeits with packaging similar to Type 1 counterfeit artesunate seized in Operation Jupiter.^8^ The blisterpacks did not bear any holograms, the print quality was inferior to authentic reference samples and they bore incorrect Chinese characters “
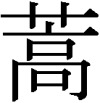
” and “
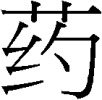
.” Two additional samples of oral artesunate, containing within range API, had differences in packaging from the available authentic samples but discussions with the manufacturer suggested that they were probably authentic.

## Discussion

This investigation highlights the frequency of counterfeit and substandard medicines in southeast Asia; that is, 32% of antimalarials and 31% of antibiotics analyzed were poor quality using the %API 85–115% range. These samples were collected as they were suspected of being poor quality and the sampling methodology does not allow these data to be used to estimate the prevalence of counterfeit and substandard medicines. However, the presence of these drugs in the market poses an important public health threat. Counterfeit and substandard essential medicines have important implications for the therapy of individual patients and control of both malaria and bacterial pathogens. The lower-than-expected API contents of counterfeit/substandard drugs are likely to be subtherapeutic and to engender pathogen drug resistance,^28^ which in turn has enormous deleterious effects on global public health.^29^

That any oral monotherapy artesunate was collected in 2009 is also alarming, as they have not been recommended by WHO for the treatment of uncomplicated falciparum malaria since 2007,^30^ and are likely to drive antimalarial artemisinin resistance.^14^,^31^ Monotherapy and poor-quality artemisinin derivatives and artemisinin-combination therapies (ACT), along with poor prescription and poor adherence, are likely to be important contributors to the recent rise of artemisinin resistance of *Plasmodium falciparum* malaria in western Cambodia.^32^ The finding of an ACT stating that it is a “2 days malaria treatment” is also of great concern as the evidence available strongly suggests that a minimum of a 3-day course is required and is contrary to WHO malaria treatment guidelines^33^ and will, again, increase mortality, morbidity, and engender drug resistance.

That 13% and 15% of antimalarials and antibiotics, respectively, were in plastic bags without labeling as to API content, batch number, and/or manufacturer information is also alarming. Such plastic bags, but containing multiple different medicines, have been described from the Thai/Burma border^34^ and the Thailand/Cambodia.^35^ These poorly prescribed pharmaceuticals risk treatment failure, poor patient adherence and engender antimicrobial resistance.

Limitations of this report include the lack of authentic reference samples available for comparison with most of the collected samples. Without these reference samples, the authenticity of drug dosage forms and packaging could not be ascertained. Despite writing to the manufacturers, we had great difficulty in obtaining authentic samples, as has been described.^28^ Greater responsiveness by the pharmaceutical industry to such legitimate requests would be very helpful. As a result of the lack of authentic reference samples, often only the %API is used for interpretation, which is insufficient to allow confident classification as counterfeit or substandard and some of those samples failing chemical assays may have been counterfeit rather than substandard. Irrespective of the origins of the poor-quality antimalarials and antibiotics, it is notable that DRAs suspicions that the collected medicines were poor quality were correct for some 1/3 of samples. The features that aroused their suspicions cannot be shared, because of the risk of assisting those counterfeiting medicines.

It was also not possible to distinguish whether those that failed the %API criteria, containing less than the stated amount of active ingredient were substandard or degraded or to detect repackaged expired drug based on %API. Hence, some of the samples classified as substandard may have been produced to good standards but degraded within the distribution chain due to poor storage. Field and laboratory mass spectrometric data of the consequences of degradation may help make this distinction and gauge their expected clinical impact. Until such information is available, it would be important for DRAs to inspect the manufacturers of failing products to determine whether the manufactured products leave the factory with appropriate %API. It is likely that those containing too much active ingredient are substandard, rather than degraded, but we cannot be certain of this without more information about decomposition of these products. There is very little real life information on the consequences of storage of antimalarials, or indeed for any medicine class, in hot tropical environments^27^,^36^ and on the chemical and physical consequences for medicines.

A further unresolved issue with such studies is that fewer tablets/capsules/vials than stated in pharmacopoeias are usually available.^28^ There also remains uncertainty as to the appropriate cutoffs for %API. We have used the 85–115% uniformity of dosage criteria but also included the more stringent criteria of 90–110%.^37^

Counterfeit drug analysis requires the analysis of both the drug dosage form and the packaging. Usually both aspects are counterfeit if the sample is counterfeit but occasionally, there are examples in which the drug dosage form is counterfeit and the packaging is genuine, or vice versa, for example, in cases where authentic dosage forms are placed in counterfeited packaging to extend the expiry date.^38^ Counterfeiters are looking for sustainable business and may manufacture counterfeit medicines with the correct type of APIs, and even the correct amount of APIs.^38^,^39^

During these operations, customs, DRAs, and police in each participating country conducted joint operations, with support from INTERPOL, WHO, and WCO and the private sectors. This helped build capacity for medicine regulation. Synergism, at the national, regional, and international levels, will be extremely important in combating trade in poor-quality medicines through facilitating vital information and intelligence exchange, leading to enhanced joint actions and ultimately improved medicine quality, and should be greatly encouraged. The WHO RapidAlert system provides an important means for enhancing these linkages.^40^

## Supplementary Material

Supplemental Table.

## Figures and Tables

**Table 1:** Summary of combined results for samples collected as part of Operations Storm I and Storm II

Country	Medicine/formulation	API results summary no. of failed samples (85–115%)/no. of samples (% failed)	Classification/notes
Cambodia	Amoxicillin po	2/16 (12.5%) Failed	−
	Ampicillin po	8/15 (53%) Failed	One contained 15% API plus amoxicillin 66.6 mg/cap, classified as counterfeit
	Penicillin V po	1/5 (20%) Failed	One dosage unknown, API content assumed
	Tetracycline po	1/14 (7%) Failed	One dosage unknown, API content assumed
	Chloramphenicol po	0/1 Failed	−
	Ciprofloxacin po	0/2 Failed	−
	Cloxacillin po	1/1 (100%) Failed	−
	Metronidazole po	0/1 Failed	−
	Artesunate po	3/10 (33%) Failed	One with API > 115%
	Artemether po	0/1 Failed	−
	DHA-piperaquine po	2/3 (67%) Failed	Wrongly labeled as “2 days malaria treatment”
	Mefloquine + artesunate po	2/7 (29%) Failed	Co-blistered mefloquine tablets missing from 2 samples and therefore no mefloquine chemical data
	Chloroquine po	1/7 (14%) Failed	1 with API > 115%. Dosages unknown, API content assumed
Lao PDR	Amoxicillin po	5/6 (83%) Failed	−
	Ampicillin po	5/5 (100%) Failed	−
	Tetracycline po	1/2 (50%) Failed	API > 115%
	Co-trimoxazole po	4/4 (100%) Failed	Great variations in sulfamethoxazole content within blisters—all out of 85–115% API; all four with trimethoprim < 85%
	Ciprofloxacin po	1/4 (25%) Failed	−
	Artesunate po	2/14 (14%) Failed	One counterfeit (no API with Type 1 fake packaging)
	Artesunate iv/im	0/1 Failed	−
	Quinine po	9/10 (90%) Failed	Dosages unknown, API content assumed
	Quinine iv	0/1 Failed	−
	Chloroquine po	1/1 (100%) Failed	API > 115%
	Sulfadoxine-pyrimethamine po	3/3 (100%) Failed	One with sulfadoxine > 115%
Indonesia	Artesunate iv/im	0/2 Failed	−
	Artemether im	0/1 Failed	−
	Artesunate + amodiaquine po	1/4 (25%) Failed	−
Vietnam	Artesunate po	1/5 (20%) Failed	−
	Chloroquine po	0/3 Failed	−
	Quinine po	0/1 Failed	−
	Sulfadoxine-pyrimethamine po	0/3 Failed	−
Myanmar/Burma	Artesunate po	1/4 (25%) Failed	−
	Artemether po	0/4 Failed	−
	Mefloquine po	0/1 Failed	−
	Mefloquine + artesunate po	0/1 Failed	−
Thailand	Doxycycline po	0/1 Failed	−
	Tetracycline po	0/8 Failed	Four dosages unknown, API content assumed
	Penicillin V po	0/7 Failed	One dosage unknown, API content assumed
	Artesunate po	0/1 Failed	−
	Artesunate iv	0/1 Failed	−
	Chloroquine po	3/5 (60%) Failed	One dosage unknown, API content assumed
	Quinine po	1/1 (100%) Failed	API > 115%, dosage unknown, API content assumed

API = active pharmaceutical ingredient; DHA = dihydroartemisinin; iv = intravenous; im = intramuscular; po = per oral.
