# Erratum

**DOI:** 10.4269/ajtmh.934err

**Published:** 2015-10-07

In *Am J Trop Med Hyg 92 (6 Suppl):* 105–112 by Yong and others, the captions for figures 2 and 3 are reversed. The journal regrets this error.

