# Supplementary material for: Collaborative Health and Enforcement Operations on the Quality of Antimalarials and Antibiotics in Southeast Asia
Source: Am J Trop Med Hyg. 2015 Jun 3;92(Suppl 6):105–12. doi: 10.4269/ajtmh.14-0574 (PMC4455084; doi:10.4269/ajtmh.14-0574)
Supplement: Supplementary file 1 [file SD6.pdf]

Supplemental Table 1

Analysis of samples collected as part of Operations Storm I and Storm II

Country/source	Medicine/mg per unit	No. of failed samples (90–110%)/ No. collected (% failed)	%API details for failed samples	Notes and packaging	Seizure location (sample ID)	Availability of authentic reference sample	Classification
Operation Storm 1							
Antibiotics							
Cambodia	Oral amoxicillin/250 mg/cap	1/3 (33%)	87.2	Samples (1) that failed API content:			
				One unit labeled as “AMOXICILLIN CAPSULES BP 250 mg” by “Glenmark,” batch number AJ4498004.	Siem Reap	×	PQ < 90%
				Samples (2) that passed API content:			
				One unit labeled as “AMOX - MS 250 Amoxillin 250 mg” by “MS,” batch number 87299.	Siem Reap	✓	Authentic
				One unit labeled as “Rx HAGIMOX, Amoxicilin 250 mg Nhai” by “CTCP DUOC HAU GIANG.” Batch number not stated.	Preah Vihear	×	90–110%
Cambodia	Oral amoxicillin/500 mg/cap	2/5 (40%)	89.6	Samples (2) that failed API content:			
				One unit labeled as “Amoxicillin 500 mg,” by “Laboratoires Ephac Co. Ltd,” batch number 82024.	Siem Reap	×	PQ < 90%
			81.1	One unit labeled as “AMOXICO(TM)-500” by “Plethico Pharmaceuticals Limited,” batch number 06533.	Preah Vihear	×	PQ < 85%
				Samples (3) that passed API content:			
				Two units labeled as “Amoxicillin 500 mg” by “BAILLY-CREAT,” batch number 132 and 125.	Siem Reap (132); Phnom Penh (125).	✓	Authentic
				One unit labeled as “AMOX-MS 500, Amoxicillin 500 mg” by “MS,” batch number 87110.	Phnom Penh	✓	Authentic
				One unit labeled as “Pulmoxyl (R)-500 AMOXICILLIN CAPSULES BP 500 mg” by “Micro Laboratories Limited,” batch number “PXFB0327”	Siem Reap	×	90–110%
Cambodia	Oral ampicillin/500 mg/cap	3/7 (43%)	–	Samples (3) that failed API content:			
			45.8	One unit labeled as “AMPICILLIN, Ampicillin 500” by “Shijiazhuang Ouyi Pharmaceutical Co., Ltd,” batch number 0529	Siem Reap	×	PQ < 85%
			89.5	One unit labeled as “AMPICILLIN Capsules BP 500 mg” by “Glenmark,” batch number “AJ4368019.”	Siem Reap	×	PQ < 90%
			84.0	One unit labeled as “AMPICO-500, Ampicillin, Gelules B.P. 500 mg” by “Plethico Pharmaceuticals Ltd,” batch no. 6393	Preah Vihear	×	PQ < 85%

(continued)

Supplemental Table 1
Continued

Operation Storm 1

Country/source	Medicine/mg per unit	No. of failed samples (90–110%)/ No. collected (% failed)	%API details for failed samples	Notes and packaging	Seizure location (sample ID)	Availability of authentic reference sample	Classification
			–	Samples (4) that passed API content:			
			–	One unit labeled as "AMPICILLINE 500 mg" by "Bailly-Creat," batch number 295.	Siem Reap	×	90–110%
			–	One unit labeled as "AMPICILLIN CAPSULES BP 500 mg, Dynacillin-500" by "Micro Laboratories Limited," batch number DYFB0039.	Siem Reap	×	90–110%
			–	One unit labeled as "AMPICILLINE 500 mg" by "LABORATOIRES EPHAC Co., Ltd," batch number 82013.	Siem Reap	×	90–110%
			–	One unit labeled as "AMPIMASTER, Ampicillin Capsules 500 mg" by "Shandong Reyoung Pharmaceutical Co., Ltd," batch number 080808.	Preah Vihear	×	90–110%
Cambodia	Oral phenoxymethylpenicillin (Pen V)/~0.6 g/tab	0/1 (0%)	–	One unit labeled as "Ospen® 1000" Phenoxymethylpenicillin as potassium salt B.P 1,000,000 IU "(~0.6 g)" by "SANDOZ," batch number 152890.	Unknown	×	90–110%
Cambodia	Oral phenoxymethylpenicillin (Pen V)/mg/tab not stated. Assumed to be 250 mg/tab	0/1 (0%)	–	Loose tablets packed in plastic bag without identity labeling.	Phnom Penh	×	90–110%
Cambodia	Oral phenoxymethylpenicillin (Pen V)/250 mg/tab	0/1 (0%)	–	One unit with paper slip stating "Penicillin-V Tablet, Penicillin V Potassium equivalent to Penicillin V 250 mg. (400,000 iu), 60 tablets" by "T. Man Pharma Ltd, Part.," batch number 6025104.	Preah Vihear	×	90–110%
Cambodia	Oral tetracycline/250 mg/cap	0/3 (0%)	–	One unit with paper slip stating "TETRAMAN CAPSULE, Tetracycline HCl 250 mg" by "T.Man Pharma Ltd, Part.," batch number 7455106.	Preah Vihear	×	90–100%
				One unit labeled as "TETRA-250, Tétracycline HCl 250 mg" by "Labo.EPHAC," batch number 81066.	Preah Vihear	×	90–110%
				One unit labeled as "TETRA-250, Tetracycline Hydrochloride 250 mg" by "EPHAC Co., Ltd," batch number 81098.	Preah Vihear	×	90–110%
Antimalarials							
Cambodia	Oral artesunate/50 mg/tab	1/5 (20%)	83.4	Samples (1) that failed API content:			
				One unit labeled as "Artesunate 50 mg" by "Binhdinh Pharma," batch number 02706.	Phnom Penh	×	PQ < 85%

(continued)

Supplemental Table 1
Continued

Country/source	Medicine/mg per unit	No. of failed samples (90–110%)/ No. collected (% failed)	%API details for failed samples	Notes and packaging	Seizure location (sample ID)	Availability of authentic reference sample	Classification
Operation Storm 1							
				Samples (4) that passed API content:			
				Two units labeled as "青蒿琥酯片, Artesunate Tablets, 50 mg × 12 片, 50 mg × 12 tablets, 桂林南药股份有限公司" by "Guilin Pharmaceutical Co., Ltd China," batch number 071201	Olympic Market, Phnom Penh	✓	Authentic
				Five units labeled as "Artesunate 50 mg" by "Binhdinh Pharma," three with batch number 2307, one with batch number 0108 and one with batch number 6508.	Rattanakiri (2307); Olympic Market, Phnom Penh (0108); Siem Reap (6508).	×	90–100%
Cambodia	Oral chloroquine/250 mg/tab	0/3 (0%)	–	One unit consisted of loose tables packed in plastic bag indicated as manufactured by "Adhon," batch number 9305 by the submitter.	Preah Vihear	×	90–110%
				One unit consisted of loose tables packed in plastic bag indicated as "Chloroquine 250 mg," batch number 216AM by the submitter. Manufacturer not stated.	Preah Vihear	×	90–110%
				One unit consisted of loose tables packed in plastic bag indicated as "Chloroquine" by "ACDHO" by the submitter. Batch number not stated.	Siem Reap	×	90–110%
Cambodia	Oral dihydroartemisinin (40 mg)-piperaquine phosphate (320 mg) co-formulated tabs	0/1 (0%)	–	One unit labeled as "Artekin, Compound Dihydroartemisinin" by "Holleykin Pharmaceutical Co., Ltd," batch number 011206.	Olympic Market, Phnom Penh	× s	90–110%
Cambodia	Oral mefloquine (250 mg) + artesunate (50 mg) co-blistered tabs	0/3 (0%)	–	Three samples labeled as "A + M2," "A + M3" and "A + M4" with batch numbers 0818, 00718 and 303117, respectively. Manufacturer not stated.	Kandal	×	90–110%
Cambodia	Oral mefloquine (250 mg) + artesunate (200 mg) co-blistered tabs	1/2 (50%)	Artesunate 85.0, Mefloquine 103.1	Sample (1) that failed API content:			
				One labeled as "Malarine (R), 3 Day Malaria Treatment, COMPOSITION: Mefloquine (250 milligrams base) 4 tablets, Artesunate (200 mg base) 3 tablets," "product imported and repackaged in Cambodia," batch number 337116.	Phnom Penh	✓	Substandard < 90%
				Sample (1) that passed API content:			
				One labeled as "Malarine" (Thai Printing), batch number 02018. Manufacturer not stated.	Dumex Market, Phnom Penh	✓	Authentic
Indonesia	im artemether/80 mg/vial	1/1 (100%)	88.5	One unit labeled as "Artemether for injection" by "Kunming Pharmaceutical Corp.," batch number 20050630.	Jayapura, Papua	✓	Substandard < 90%

(continued)

Supplemental Table 1
Continued

Country/source	Medicine/mg per unit	No. of failed samples (90–110%)/ No. collected (% failed)	%API details for failed samples	Notes and packaging	Seizure location (sample ID)	Availability of authentic reference sample	Classification
Operation Storm 1							
Indonesia	iv/im artesunate/60 mg/vial	0/2 (0%)	–	Two samples labeled as "Artesunate for injection" by "Guilin Pharmaceutical Co., Ltd, China," batch number ZA20060905 and ZA071102.	Ambon, Maluku (ZA20060905); Jayapura, Papua (ZA071102)	✓	Authentic
Indonesia	Oral artesunate (50 mg) + amodiaquine (153 mg) co-blistered tabs	1/2 (50%)	Artesunate 79.8, Amodiaquine 98.2	Sample (1) that failed API content:			
				One unit labeled as "Artesdiaquine (R) tablets, Artesunate 50 mg × 12 Tabs + Amodiaquine 200 mg × 12 Tabs" by "Beijing Wanhui Double Crane Pharmaceutical Co., Ltd, Beijing, China," batch number 030406.	Jayapura, Papua	×	PQ < 85%
				Sample (1) that passed API content:			
				One unit labeled as "Artesdiaquine (R) tablets, Artesunate 50 mg × 12Tabs + Amodiaquine 200mg × 12Tabs" by "Beijing Wanhui Double Crane Pharmaceutical Co., Ltd, Beijing, China," batch number 091006.	Ambon, Nusa Tenggara Timur	×	90–110%
Indonesia	Oral artesunate (50 mg) + amodiaquine (150 mg) co-blistered tabs	0/2 (0%)	–	Two samples labeled as "ARSUAMOON, Artesunate Tablet + Amodiaquine Hydrochloride Tablets" by "Guilin Pharmaceutical Co., Ltd, China," batch numbers L0071110 and L0071105.	Jayapura, Papua (L0071110); Ambon, Nusa Tenggara Timur (L0071105).	×	90–110%
Lao PDR	Oral artesunate/50 mg/tab	4/9 (44%)	–	Samples (4) that failed API content:			
			0, 0	Two units labeled as "青蒿琥酯片, Artesunate Tablets, 50 mg × 12 片, 50 mg × 12 tablets, 桂林南药股份有限公司" ("Guilin Pharmaceutical Co., Ltd, China"), batch number 060207. The blister packs did not bear any holograms and had Type 1 counterfeit packaging.	Southern Laos	✓	Counterfeit
			86.2	One unit labeled as "Artesunat 50 mg" by "Mekophar," batch number 06001AN.		✓	Substandard < 90%
			143.5	One unit labeled as "Artesunat 50 mg" by "Mekophar," batch number 06001AN.		✓	Substandard > 115%
			89.6	One unit labeled as "Artesunate 50 mg" by "Mediplantex," batch number 061106		✓	Substandard < 90%
			–	Samples (5) that passed API content:			
			–	One unit labeled as "Artesunat 50 mg" by "Nam Ha (Naphaco)," batch number 07102.		✓	Authentic
			–	Two samples labeled as "Artesunat 50 mg" by "Mekophar," batch number 07006FN and 06004AN.		✓	Authentic
			–	One unit labeled as "Artesunate 50 mg" by "Mediplantex," batch number 39007.		×	90–110%
			–	One unit labeled as "Artesunat 50 mg" by "X.N.D.P.T.U.I," batch number 010807.		x	90–110%

(continued)

Supplemental Table 1
Continued

Country/source	Medicine/mg per unit	No. of failed samples (90–110%)/ No. collected (% failed)	%API details for failed samples	Notes and packaging	Seizure location (sample ID)	Availability of authentic reference sample	Classification
Operation Storm 1							
Myanmar	Oral artemether/50 mg/tab	0/4 (0%)	–	Three samples labeled as “Antimalarial, Artem, Artemether Tablets ART 50 mg” by “Kunming Pharmaceutical Corp., China,” batch numbers 06116801, 070DF2 and 07DF1.	Bahan, Yangoon (06116801); Myawaddy (070DF2, 07DF1).	×	90–110%
				One unit labeled as “AA-Artemether 50 mg Antimalarial Agent, ART, 50 mg” by “AA Medical Products Ltd,” batch number 070911.	Kawthaung	×	90–110%
Myanmar	Oral artesunate/50 mg/tab	1/4 (25%)	79.9	Samples (1) that failed API content:			
				One unit labeled as “AA-Artesunat 50 mg, 12 tablets, Antimalarial Agent” by “AA Medical Products Ltd,” batch number 070901.	Bahan, Yangoon	×	PQ < 85%
				Samples (3) that passed API content:			
				Two samples labeled as “Specific antimalarial, ARTESUNATE TABLETS, 青蒿琥酯片, 桂林南药股份有限公司” by “Guilin Pharmaceutical Co., Ltd,” batch numbers 070908 and 080206. Some differences from authentic sample but concluded to be probably authentic sample.	Myawaddy	✓	Authentic
				One unit labeled as “ORAL ROUTE ARTESUNAT” by “Central Pharmaceutical Factory,” batch number 010607.	Tachileik	×	90–110%
Myanmar	Oral mefloquine/250 mg/tab	0/1 (0%)	–	One unit labeled as “Mevax, Mefloquine Tablets 250 mg” by “Ajanta pharma limited,” batch number M0037D.	Tachileik	×	90–110%
Myanmar	Oral mefloquine (250 mg) + artesunate (200 mg) co-blistered tabs	1/1 (100%)	Artesunate 91.9, Mefloquine 88.1	One unit labeled as “Arfloquin” by “Mekophar,” batch number 07003FX.	Kawthaung	×	Mefloquine PQ < 90%
Thailand	iv artesunate/60 mg/vial	1/1 (100%)	110.9	One unit labeled as “Artesunate for injection” by “Guilin Pharmaceutical Co., Ltd, Guangxi, China,” batch number ZA080202. (Imported by Atlantic Pharmaceutical Co., Ltd, Bangkok, Thailand).	Unknown	×	PQ > 110%
Thailand	Oral artesunate/50 mg/tab	0/1 (0%)	97.2%	One unit labeled as “青蒿琥酯片, Artesunate Tablets” by “桂林南药股份有限公司” (“Guilin Pharmaceutical Co., Ltd, China”), batch number 080207. Some differences from authentic sample but concluded to be probably authentic sample.	Unknown	✓	Authentic
Vietnam	Oral artesunate/50 mg tab	1/5 (20%)	83.7	Samples (1) that failed API content:			
				One labeled as “Artesunat 50 mg” by “Mekophar Chemical Pharmaceutical	Hanoi city	✓	Substandard < 85%

(continued)

Supplemental Table 1
Continued

Operation Storm 1

Country/source	Medicine/mg per unit	No. of failed samples (90–110%)/ No. collected (% failed)	%API details for failed samples	Notes and packaging	Seizure location (sample ID)	Availability of authentic reference sample	Classification
				Joint-Stock Co. HCM, Vietnam," batch number 07001FN.			
				Samples (4) that passed API content:			
				One unit labeled as "Artesunat 50 mg" by "CTCPDP Nam Ha (Naphaco)," batch number 07101.	Vinh Loc District, Thanh Hoa Province	✓	Authentic
				Two samples labeled as "Artesunat 50 mg" by "CTCPHDP Mekophar" with batch number 06001AN and 06004AN.	Ha Giang Town, Ha Giang Province (06001AN); Unknown (06004AN)	✓	Authentic
				One unit labeled as "Artesunat 50 mg" by "Mekophar Chemical Pharmaceutical Joint-Stock Co. HCM, Vietnam" with batch number 07004FN.	Thanh Hoa Town, Thanh Hoa Province	✓	Authentic
Vietnam	Oral chloroquine/250 mg/tab	0/3 (0%)	–	One unit consisted loose tablets packed in plastic bag with paper label stating "Chloroquine 250 mg" by "Mekophar Chemical Pharmaceutical Joit-Stock Co," lot number "06001AN" and expiry date "06/2010"	Ha Giang Town, Ha Giang Province	×	90–110%
				Two units labeled as "CLOROQUIN phosphat 250 mg" by "Namha Pharmaceutical Joint-Stock Co, Vietnam," batch number 06102.	Ha Giang Town, Ha Giang District	✓	Authentic
				One unit labeled as "CLOROQUIN PHOSPHAT 250 mg" by "Mekophar, HCM City," batch number 07001CN.	Ha Noi city	✓	Authentic
Vietnam	Oral quinine/250 mg/tab	0/1 (0%)	–	Consisted loose tablets packed in plastic bag with paper label stating "Quinine 250 mg" by "Mekophar Chemical Pharmaceutical Joit-Stock Co," batch number "0030905"	Ha Giang Town, Ha Giang Province	×	90–110%
Vietnam	Oral sulfadoxine (500 mg) + pyrimethamine (25 mg) co-formulated tabs	0/3 (0%)	–	Three samples labeled as "CTCP Hoa-Duoc pham, Mekophar, MEKOFAN" by "Mekophar," batch numbers 0010405, 0021205, and 06001AN.	Vinh Loc District, Thanh Hoa Province	×	90–110%

Operation Storm II

Country/source	Medicine/mg per unit	No. of failed samples (90–110%)/ no. collected (% failed)	Chemical assay details for failed samples (actual %API)	Notes and packaging	Seizure location	Authentic packaging and dosage forms available	
Antibiotics							
Cambodia	Oral amoxicillin/250 mg/cap	1/1 (100%)	85.3	One unit labeled as "Amoxicillin 250 mg" by "LABORATOIRES EPHAC Co., Ltd," batch number 82030.	Unknown	×	PQ < 90%
Cambodia	Oral amoxicillin/500 mg/cap	1/7 (14%)	48.5	Sample (1) that failed API content:			
				One unit labeled as "AMOXICILLIN 500 mg" by "Shijiazhuang Ouyi	Unknown	×	PQ < 85%

(continued)

Supplemental Table 1
Continued

Country/source	Medicine/mg per unit	No. of failed samples (90–110%)/ no. collected (% failed)	Chemical assay details for failed samples (actual %API)	Notes and packaging	Seizure location	Authentic packaging and dosage forms available	
Operation Storm II							
				Pharmaceutical Co., Ltd," batch number 0329.			
				Samples (6) that passed API content:			
				One unit labeled as "AMOXICILLINE 500 mg" by "Laboratoires B.F.," batch number 83.	Pailin	×	90–110%
				One unit labeled as "AMOXICILLIN CAPSULES BP 500 mg" by "E.K.H Co., Ltd," batch number 081110.	Unknown	×	90–110%
				One unit labeled as "Amoxicillin 500 mg" by "Mekophar," batch number 09005AN.	Unknown	×	90–110%
				One unit labeled as "AMOXMASTER, Amoxicillin Capsules 500 mg" by "SHANDONG Reyoung PharmaceuticaL Co., Ltd," batch number 080807	Unknown	×	90–110%
				One unit labeled as "AMOXICILLINE 500 mg" by "Laboratories P.P.M.," batch number 1027	Unknown	×	90–110%
				One unit labeled as "AMOXICILLIN CAPSULES BP 500 mg" by "Glenmark," batch number AE4377019.	Unknown	×	90–110%
Cambodia	Oral ampicillin/250 mg/cap	1/1 (100%)	70.4	One unit labeled as "AMPICILLINE 250 mg" by "Laboratoires Ephac Co.Ltd," batch number 82009.	Unknown	×	PQ < 85%
Cambodia	Oral ampicillin/500 mg/cap	5/7 (71%)	–	Samples (5) that failed API content:			
			42.3	One unit labeled as "AMPICILLIN 500 mg" by "Shijiazhuang Ouyi PharmaceuticaL Co., Ltd" with batch number 0529.	Unknown	×	PQ < 85%
			74.8	One unit labeled as "AMPICILLIN Capsules BP 500 mg" by "Glenmark" with batch number AJ4368028.	Unknown	×	PQ < 85%
			71.1	One unit labeled as "AMPICILLIN CAPSULES B.P. 500 mg" by "Flamingo Pharmaceuticals Ltd," batch number 413.	Stung Treng	×	PQ < 85%
			80.6	One unit labeled as "AMPICILLIN CAPSULES B.P. 500 mg" by "E.K.H Co., Ltd," batch number 081109M.	Unknown	×	PQ < 85%
			15.3 (contains amoxicillin 66.6 mg/cap)	One unit labeled as "AMPIMEX-500, Ampicillin Capsules B.P.500 mg," by "Fu Li Pharmaceutical, China." Batch number not stated.	Battambang	×	Counterfeit
			–	Samples (2) that passed API content:			
			–	One unit labeled as "AMPIMASTER, Ampicillin Capsules 500 mg" by "Shandong Reyoung Pharmaceutical Co., Ltd," batch number 080808.	Battambang	×	90–110%

(continued)

SUPPLEMENTAL TABLE 1
Continued

Operation Storm II							
Country/source	Medicine/mg per unit	No. of failed samples (90–110%)/ no. collected (% failed)	Chemical assay details for failed samples (actual %API)	Notes and packaging	Seizure location	Authentic packaging and dosage forms available	
			–	One unit labeled as "AMPI-MS 500, Ampicillin 500 mg" by "Medical Supply Pharmaceutical Enterprise (MS)," batch number IT97095.	Koh Kong Province	×	90–110%
Cambodia	Oral chloramphenicol/ 250 mg/cap	0/1 (0%)	–	One unit labeled as "CHLORAMPHENICOL" by "Hovid," batch number AH10598.	Unknown	×	90–110%
Cambodia	Oral ciprofloxacin/500 mg/tab	0/2 (0%)	–	One unit labeled as "CIPROXIN-500, Ciprofloxacin Tablets USP 500 mg." by "Umedica Laboratories Pvt Ltd," batch number ZB801.	Kampong Cham	×	90–110%
				One unit labeled as "ECOFLOX-500, CIPROFLOXACIN TABLETS BP" by "Medley Pharmaceuticals Ltd," batch number D80244.	Unknown	×	90–110%
Cambodia	Oral cloxacillin/500 mg/cap	1/1 (100%)	55.1	One unit labeled as "CLOXA-MS 500" by "Medical Supply Pharmaceutical Enterprise (MS)" with batch number 97014.	Koh Kong Province	×	PQ < 85%
Cambodia	Oral metronzidazole/250 mg/tab	0/1 (0%)	–	One unit labeled as "Metronidazole Tablets B.P.250 mg" by "Lyka Laboratories Limited," batch number TL-137E.	Kampong Cham	×	90–110%
Cambodia	Oral phenoxymethylpenicillin (Pen V)/400,000 IU/tab	0/1 (0%)	–	One unit labeled as "PENICILLIN V PENICILLIN V POTASSIUM equi. to PENICILLIN V 400,000 units" by "T.Man Pharma Ltd, Part." with batch number L/C6015102.	Ming Prov. Psar Pahi	×	90–110%
Cambodia	Oral phenoxymethylpenicillin (Pen V)/500,000 IU/tab	1/1 (100%)	82.5	One unit labeled as "SQUASH - PHENOXYMETHYL PENICILLIN 500,000 IU" by "Chea Chamnan Laboratoire Co., Ltd," batch number 080511.	Unknown	×	PQ < 85%
Cambodia	Oral tetracycline/250 mg/tab or cap	2/7 (29%)	–	Samples (2) that failed API content:			
			81.7	One unit labeled as "BIOMAN, Sugar coated tablet, Tetracycline HCl 250 mg" by "T. Man Pharma Ltd, Part." Batch number not stated.	Unknown	×	PQ < 85%
			87.4	One unit labeled as "抗毒消炎丸, AQ, Masa Laboratory, CONFIT, Tetracycline Hydrochloride Capsule 250 mg." by "Masa Laboratory," batch number 610245.	Unknown	×	PQ < 90%
			–	Samples (5) that passed API content:			
			–	Two samples labeled as "Tetraman, Tetracycline Hydrochloride Capsules 250 mg, 抗毒消炎丸" by "T.Man Pharma Ltd, Part," batch number L/C7455106 and L/C7455108.	Unknown (L/C7455106); Kampong Cham (L/C7455108)	×	90–110%
			–		Unknown	×	90–110%

(continued)

Supplemental Table 1
Continued

Country/source	Medicine/mg per unit	No. of failed samples (90–110%)/ no. collected (% failed)	Chemical assay details for failed samples (actual %API)	Notes and packaging	Seizure location	Authentic packaging and dosage forms available	
Operation Storm II							
				One unit labeled as “TETRA-250, Tétracycline HCl 250 mg” by “Labo.EPHAC,” batch number 81099.			
			–	One unit labeled as “抗毒消炎丸Vescomycin Tetracycline Hydrochloride 250 mg” by “VESCO,” batch number 151013.	Unknown	×	90–110%
			–	One unit labeled as “抗毒消炎丸, C.M.P, Chea Chamnan Laboratoire, TETRA C.M.P, Tetracycline Hydrochloride 250 mg. Capsule” by “Chea Chamnan Laboratoire Co., Ltd,” batch number 081009.	Unknown	×	90–110%
Cambodia	Oral tetracycline/500 mg/cap	1/3 (33%)	88.5	Sample (1) that failed API content:			
				One unit labeled as “TETRACYCLINE 500 mg” by “LABO.EPHAC,” batch number 91010.	Koh Kong Province	×	PQ < 90%
				Samples (2) that passed API content:			
				One unit labeled as “TETRACYCLINE 500 mg” by “Labo.Ephac,” batch number 81136.	Phnom Penh	×	90–110%
				One unit labeled as “TETRA-MS 500 Tetracycline 500 mg” by “MS,” batch number 80352.	Phnom Penh	×	90–110%
Cambodia	Oral tetracycline/mg/cap not stated. Assumed to be 250 mg/cap	1/1 (100%)	85.9% (214.81 mg/cap)	One unit consisted of loose capsules packed in plastic bag without identity labeling. Capsules were printed with “MS” logo. Batch number not stated.	Unknown	×	PQ < 90%
Lao PDR	Oral sulfamethoxazole (400 mg)-trimethoprim (80 mg) co-formulated tab #	4/4 (100%)	Sulfamethoxazole (124,123,81,83,82), (112,80,81), (109,81,82,82), (114,117,80,83,82), Trimethoprim (84,82,96,98,96), (66,76,76), (65,87,92,97) and (69,67,84,87,86)	Samples (4) that failed API content:			
				One unit as “Srim-side” by “KPN Pharma Factory,” Lot 904002	Vientiane Capital	×	PQ < 85%<br>> 115%
				One unit labeled as “Sulfatrim,” Lot 0811035		×	PQ < 85%<br>> 115%
				Two samples labeled as “Vactrim” by “Codupha-Lao Pharma Factory,” Lot 916302 and 816303		×	PQ < 85%<br>> 115%
Lao PDR	Oral amoxicillin/500 mg/cap	5/6 (83%)	82.2, 82.6, 84.1, 75.7 and 86.8	Samples (5) that failed API content:			
				Five labeled as “CODUPHA-LAO, AMOXYCILLIN, Amoxycilline 500 mg” by “Codupha.” Four failed API% 85–115% - one with batch number 800252, three with batch number 800251 and one failed API% 90–110% with batch number 800234.	Vientiane Capital	×	PQ < 85%

(continued)

Supplemental Table 1
Continued

Operation Storm II							
Country/source	Medicine/mg per unit	No. of failed samples (90–110%)/ no. collected (% failed)	Chemical assay details for failed samples (actual %API)	Notes and packaging	Seizure location	Authentic packaging and dosage forms available	
				Samples (1) that passed API content: One labeled as "Amoxy M.H., Amoxicillin (as trihydrate) 500 mg" by "M and H Manufacturing Co., Ltd" with batch number 23CZ.		×	90–110%
Lao PDR	Oral ampicillin/250 mg/tab	1/1 (100%)	67.1	Loose tablets packed in ziplock bags with paper label stating "Ampicilline." Manufacturer and batch number were not stated.	Vientiane Capital	×	PQ < 85%
Lao PDR	Oral ampicillin/500 mg/cap	4/4 (100%)	79.0, 67.7 82.9, 79.1, 83.3 61.6	Two samples labeled as "CODUPHA-LAO, AMPICILLIN, Ampicillin 500 mg" by "Codupha" with batch number 800961 and 800945.	Vientiane Capital	×	PQ < 85%
				Three units labeled as "AMPICILLIN 500, Ampicillin Trihydrate 500 mg" by "P.D.C.," batch number 012.		×	PQ < 85%
				One unit labeled as "AMPICILLINE CAPSULES" by "Zhangfeng Pharmaceutical Factory, Longchuan, Yunnan, China," batch number 081101.		×	PQ < 85%
Lao PDR	Oral ciprofloxacin/500 mg/tab	1/4 (25%)	79.0	Sample (1) that failed API content: One unit labeled as "Ciprocin 500," batch number 021007. Manufacturer was not stated.	Vientiane Capital	×	PQ < 85%
				Samples (3) that passed API content: One unit labeled as "Ciprofloxacin, Ciprofloxacin HCl 500 mg," batch number 28241207. Manufacturer was not stated.	Vientiane Capital	×	90–110%
				One unit labeled as "CIPROFLOXACIN Tablets U.S.P. 500 mg" by "Brawn Laboratories Ltd," batch number BTG18017.		×	90–110%
				One unit labeled as "CIPROFLOXACIN 500 mg" by "Thephaco," batch number 050908.		×	90–110%
Lao PDR	Oral tetracycline/250 mg/cap	1/1 (100%)	89.6	One unit labeled as "HEROLMYCIN, 250 mg, Capsule" by "KPN Pharma Factory," batch number 801007.	Vientiane Capital	×	PQ < 90%
Lao PDR	Oral tetracycline/250 mg/tab	1/1 (100%)	131.3	One unit consisted of loose tablets packed in plastic bag labeled as "Leomycine Tab 250 mg, 072011." Manufacturer and batch number not stated.	Vientiane Capital	×	PQ > 115%
Thailand	Oral doxycycline/100 mg/tab	0/1 (0%)	–	One unit labeled as "Doxycycline Hyclate Tablets," USP 100 mg by "West-ward Pharmaceutical Corp.," batch number 66112A.	Suvannabumi Terminal Airport, Bangkok	×	90–110%

(continued)

Supplemental Table 1
Continued

Operation Storm II

Country/source	Medicine/mg per unit	No. of failed samples (90–110%)/ no. collected (% failed)	Chemical assay details for failed samples (actual %API)	Notes and packaging	Seizure location	Authentic packaging and dosage forms available	
Thailand	Oral phenoxymethylpenicillin (Pen V)/250 mg/tab	0/1 (0%)	–	One unit consisted loose tablets packed in plastic bag without identity labeling. Indicated as “Metro-V 250” with batch number 0104022 by the submitter. Manufacturer was not stated.	Maesod District, Tak	×	90–110%
Thailand	Oral phenoxymethylpenicillin (Pen V)/400,000 IU/tab	0/2 (0%)	–	One unit consisted loose tablets packed in plastic bag labeled as “Pen V. 400,000.” Indicated as manufactured by “Unison Laboratories Co., Ltd” with batch number “8PH30” by the submitter.	Muang District, Ranong	×	90–110%
				One unit labeled as “PENICILLIN V POTASSIUM 400,000 I.U. (250 mg)” by “Unison Laboratories Co., Ltd,” batch number 8PQ161.		×	90–110%
Thailand	Oral phenoxymethylpenicillin (Pen V)/500,000 IU tab	0/1 (0%)	–	One unit labeled as “PENICILLIN TABLETS” by “A.N.B. Laboratories Co., Ltd,” batch number 514634.	Muang District, Ranong	×	90–110%
Thailand	Oral phenoxymethylpenicillin (Pen V)/800,000 IU tab	0/2 (0%)	–	Two samples labeled as “PENICILLIN V POTASSIUM 800,000 I.U.” by “Unison Una” (“Unison Laboratories Co., Ltd”), batch number L8PR01 and L8PR03.	Maesod District, Tak (L8PR01); Muang District, Ranong (L8PR03)	×	90–110%
Thailand	Oral phenoxymethylpenicillin (Pen V)/mg/tab not stated. Assumed to be 250 mg/tab	0/1 (0%)	–	One unit consisted of loose tablets packed in plastic bag without identity labeling. Indicated as “Penicillin V potassium” by the submitter. Manufacturer and batch number not stated.	Maesod District, Tak	×	90–110%
Thailand	Oral tetracycline/250 mg/cap	1/3 (33%)	86.6	Sample (1) that failed API content:			
				One unit consisted of loose capsules in plastic bag without identity labeling. Indicated as “Tetracycline 250 mg” with batch number 5112051 by the submitter. Manufacturer was not stated.	Maesod District, Tak	×	PQ < 90%
				Samples (2) that passed API content:			
				One unit labeled as “Tetra. 250 mg.” Indicated as manufactured by “Pharmasant Laboratories Co., Ltd” with batch number L21015 by the submitter.	Muang District, Ranong	×	90–110%
				One unit consisted of loose capsules in plastic bag without identity labeling. Indicated as manufactured by “Seven Star Co., Ltd” with batch number 0824 by the submitter.	Muang District, Ranong	×	90–110%
Thailand	Oral tetracycline/500 mg/cap	1/1 (100%)	87.3	One unit consisted of loose capsules in plastic bag without identity labeling. Indicated as “Nidacycline 500 mg” with	Maesod District, Tak	×	PQ < 90%

(continued)

Supplemental Table 1
Continued

Country/source	Medicine/mg per unit	No. of failed samples (90–110%)/ no. collected (% failed)	Chemical assay details for failed samples (actual %API)	Notes and packaging	Seizure location	Authentic packaging and dosage forms available	
Operation Storm II							
				batch number 2581052 by the submitter. Manufacturer not stated.			
Thailand	Oral tetracycline/mg/cap not stated. Assumed to be 250 mg/cap	0/4 (0%)	–	One unit consisted of loose capsules in plastic bag without identity labeling. Indicated as "NL Mycin" with batch number 104682 by the submitter. Manufacturer not stated.	Maesod District, Tak	×	90–110%
				One unit consisted of loose capsules in plastic bag without identity labeling. Indicated as "AD Mycine" with batch number C4834 by the submitter. Manufacturer not stated.	Maesod District, Tak	×	90–110%
				One unit consisted of loose capsules in plastic bag without identity labeling. Indicated as manufactured by "Asian Union Laboratories Co., Ltd" with batch number 516020 by the submitter.	Muang District, Ranong	×	90–110%
				One unit consisted of loose capsules in plastic bag without identity labeling. Indicated as "Vescomycin" by "Vesco Pharma Co., Ltd," batch number 15018 by the submitter.	Unknown	x	90–110%
Antimalarials							
Cambodia	Oral chloroquine/mg/tab not stated. Assumed to be 250 mg/tab #	1/4 (25%)	(106, 113, 111, 113), (108, 117,107, 114), (113, 116, 112, 112), 110, 147, 119, 111), (108, 122, 121, 114), (110, 122, 116, 118), (109, 118, 119, 115), (108, 114, 112, 115), (110, 115, 115, 118), (102, 113, 115, 117), (105, 114, 120, 112), (103, 115, 116, 117)	Sample (1) that failed API content:	Unknown		
				Ten units of 18–24 tablets in plastic bags w/o identity labeling, tablets with mosquito image embossed failed API% 85–115% and two units failed API% 90–110%		×	PQ > 115%
				Sample (3) that passed API content:			
				Three samples of 18–23 tablets in plastic bags w/o identity labeling, tablets with mosquito image embossed.		×	90–110%
Cambodia	Oral artesunate/50 mg/tab #	2/5 (40%)	–	Samples (2) that failed API content:			
			(88, 82, 88, 107), (84, 82, 90, 89)	Two units labeled as "Manufactured by Binhdinh Pharma" and Distributed by "Canapharm 2000 Inc., Edmonton, Canada," Lot No 2307.	Unknown	×	PQ < 85%
			(128, 119, 104, 103)	One unit labeled as "Arquine" "Medical Supply Pharmaceutical Enterprise" Phnom Penh, Cambodia, Lot number 80036.		×	PQ > 115%
			–	Samples (3) that passed API content:			
			–	Five units labeled as "Manufactured by Binhdinh Pharma" and Distributed by		×	90–110%

(continued)

Supplemental Table 1
Continued

Country/source	Medicine/mg per unit	No. of failed samples (90–110%)/ no. collected (% failed)	Chemical assay details for failed samples (actual %API)	Notes and packaging	Seizure location	Authentic packaging and dosage forms available	
Operation Storm II							
				"Canapharm 2000 Inc, Edmonton, Canada," Lot No 2908.			
			–	Two samples (7 units) labeled as "Arquine" "Medical Supply Pharmaceutical Enterprise" Phnom Penh, Cambodia, Lot numbers 80998 and 80036.		×	90–110%
Cambodia	Oral DHA (40 mg)-piperaquine (320 mg) co-formulated tabs #	2/2 (100%)	DHA (50, 83, 75), (59, 90, 87) and (74, 58), Piperaquine (108, 100, 111), (98, 108, 108), (107, 97)	Two samples labeled as "2 days malaria treatment" "Artekin" and "Holleykin Pharmaceutical Co. Ltd" Guangdong, China, Lot numbers 011206 and 010707. Packaging appeared to be genuine. All had ≤ 90% DHA than that stated, and one had piperaquine > 110%	Unknown	✓	Substandard < 85%
Cambodia	Oral artesunate (200 mg) + mefloquine (250 mg) co-blistered tabs #	1/1 (100%)	Artesunate 81 and 124, Mefloquine 108%.	One unit labeled as "Malarine" Lot 337116. Artesunate tabs embossed "Mepha."	Unknown	×	Artesunate PQ < 85% > 115%
Cambodia	Oral artesunate (50 mg) + mefloquine (unknown) co-blistered tabs #	1/1 (100%)	Artesunate (90, 87, 93, 83) and (86, 88, 93, 84)	Two units labeled as "A + M4" Lot 00618. Mefloquine tablets were missing.	Unknown	×	Artesunate PQ < 85%
Cambodia	Oral artemether/40 mg/cap #	0/1 (0%)	–	One unit labeled as "Kunming Pharmaceutical Co. Ltd," Lot 07GE1.	Unknown	✓	Authentic
Lao PDR	Oral chloroquine phosphate/ 250 mg/tab #	1/1 (100%)	Chloroquine (107, 117, 116, 116%)	Four units labeled as "MALACIN" made by "A.N.B. Laboratories Co. Ltd," Lot No. 500751. Packaging appeared genuine.	Unknown	✓	Substandard > 115%
Lao PDR	Oral quinine sulfate/mg/tab not stated. Assumed to be 250 mg/tab #	9/10 (90%)	Quinine (95, 61, 74, 70), (86, 71, 74, 81), (83, 77, 72, 75), (71, 70, 61, 71), (70, 62, 75, 71), (69, 76, 80, 81), (80, 75, 77, 76), (79, 75, 72, 77), (75, 80, 83, 84)	Samples (9) that failed API content: Nine sets of 19–22 white tablets in plastic bags w/o identity labeling. Batch number not stated. Stated dose assumed. A	Unknown	×	PQ < 85%
				Sample (1) that passed API content: One unit of 22 white tablets in plastic bags w/o identity labeling. Stated dose assumed		×	90–110%
Lao PDR	Oral sulfadoxine (500 mg)-pyrimethamine (25 mg) coformulated tabs #	3/3 (100%)	Sulfadoxine (95, 105, 94), (139, 174) Pyrimethamine (84, 45, 47), (94, 87)	Two samples labeled as "Mekofan" made by "CTCP Hoa-Duoc pharm Mekophar." Lot numbers 0021104, 0010405.	Unknown	×	PQ Pyrim < 85% Sulfa > 115%
			Sulfadoxine (76, 88, 89), (72, 89, 56) Pyrimethamine (84, 45, 46), (84, 43, 24)	Two units labeled as "Tancida" (2). Lot number or expiry date 04 12.		×	PQ Pyrim < 85% Sulfa < 85%

(continued)

Supplemental Table 1
Continued

Operation Storm II

Country/source	Medicine/mg per unit	No. of failed samples (90–110%)/ no. collected (% failed)	Chemical assay details for failed samples (actual %API)	Notes and packaging	Seizure location	Authentic packaging and dosage forms available	
Lao PDR	Oral artesunate/50 mg/tab #	1/5 (20%)	(115, 114, 113)	Sample (1) that failed API content:	Unknown		
				One sample labeled as “Artesunat” made by “Mekophar.” Lot 07006FN. Packaging appeared genuine.		✓	Substandard > 110%
				Samples (4) that passed API content:			
				Three samples (7 units) labeled as “Artesunat” made by “Mekophar.” Lot 07006FN and 09001FN, 08001FN.		✓	Authentic
				Two units labeled as made by “PHARBACO.” Lot 011207.		×	90–110%
Lao PDR	iv quinine dihydrochloride/ 600 mg/vial #	0/1 (0%)	–	One unit labeled as made by “ANB.” Exp. Date 10/2011, 07/2011, 04/2013, 04/2012.	Unknown	✓	Authentic
Lao PDR	iv artesunate (60 mg) vial and 5% sodium bicarbonate #	0/1 (0%)	–	One unit labeled as made by “Guilin Pharmaceutical Co. Ltd” Lot ZA061102 for artesunate and T061101 for bicarbonate. Bicarbonate not analyzed.	Unknown	✓	Authentic
Thailand	Oral chloroquine phosphate/ 250 mg/tab	2/4 (50%)	65.8 and 62.1	Samples (2) that failed API content:			
				One sample labeled as “CHLOROQUINE PHOSPHATE TABLETS.” Manufacturer and batch number not stated.	Maesod District, Tak	×	PQ < 85%
				One sample with loose tablets packed in plastic bag without identity labeling. Indicated as “Chloroquine phosphate 250 mg” with batch number 18139 by the submitter. Manufacturer not stated.	Maesod District, Tak	×	PQ < 85%
				Samples (2) that passed API content:			
				One sample labeled as “MALACIN” by “A.N.B. Laboratories Co., Ltd” with batch number 500751.	Muang District, Ranong	×	90–110%
				One sample labeled as “CHLOROQUINE PHOSPHATE TABLETS.” Manufacturer and batch number not stated.	Muang District, Ranong	×	90–110%
Thailand	Oral chloroquine/mg/tab not stated. Assumed to be 250 mg/tab	1/1 (100%)	71.6% (179.1 mg/tab)	Loose tablets packed in ziplocak bag labeled as “10/11/09; Chloroquine; 18079.” Manufacturer not stated.	Muang District, Ranong	×	PQ < 85%
Thailand	Oral quinine sulfate/mg/tab not stated. Assumed to be 250 mg/tab	1/1 (100%)	116.7% (291.8 mg/tab)	Loose tablets packed in plastic bag without identity labeling. Indicated as “Quinne-S” by the submitter. Manufacturer and batch number not stated.	Maesod District, Tak	×	> 115%

API = active pharmaceutical ingredient; CDC = Center for Disease Control and Prevention; DHA = dihydroartemisinin; HSA = Health Sciences Authority; PQ = poor quality (insufficient evidence to determine whether falsified or substandard).
All assayed at HSA, except # that were assayed at CDC and Georgia Tech, Atlanta, GA. Availability of authentic reference sample: ✓ available; × not available.
The criteria of 85–115% and 90–110% API have been used for interpretation. Those that failed using 85–115% API criteria are given in red. 90–110% indicates that the sample had %API within this range but authentic packaging examples were not available to allow confirmation that sample was authentic.
